# Supplementary material for: Functional arrays of human pluripotent stem cell-derived cardiac microtissues
Source: Sci Rep. 2020 Apr 24;10:6919. doi: 10.1038/s41598-020-62955-3 (PMC7181791; doi:10.1038/s41598-020-62955-3)
Supplement: Supplementary file 1 — Supplementary Info. [file 41598_2020_62955_MOESM1_ESM.docx]

Supplementary Information

**Functional arrays of human pluripotent stem cell-derived cardiac microtissues**Nimalan Thavandiran^1,2^, Christopher Hale^3^, Patrick Blit^4^, Mark L. Sandberg^5^, Michele E. McElvain^5^, Mark Gagliardi^6^, Bo Sun^6^, Alec Witty^6^, George Graham^4^, Van T.H. Do^4^, Mohsen Afshar Bakooshli^1^, Hon Le^3^, Joel Ostblom^1^, Samuel McEwen^1^, Erik Chau^7^, Andrew Prowse^4^, Ian Fernandes^6^, Andreea Norman^4^, Penney M. Gilbert^1,8-10^, Gordon Keller^6^, Philip Tagari^3^, Han Xu^5,*^, Milica Radisic^1,2,11,*^, Peter W. Zandstra^1,4,10,12,*^

^1^Institute for Biomaterials and Biomedical Engineering, University of Toronto, Toronto, Ontario, Canada.

^2^Department of Chemical Engineering and Applied Chemistry, University of Toronto, Toronto, Ontario, Canada.

^3^Amgen Discovery Research, Amgen Inc. South San Francisco, CA, USA.

^4^CCRM, Toronto, Ontario, Canada

^5^A2 Biotherapeutics Inc. Agoura Hills, CA USA

^6^McEwen Centre for Regenerative Medicine, University Health Network, Toronto, Ontario, Canada.

^7^Department of Mechanical and Industrial Engineering, University of Toronto, Toronto, Ontario, Canada.

^8^Department of Biochemistry, University of Toronto, Toronto, Ontario, Canada

^9^Cell and Systems Biology, University of Toronto, Toronto, Ontario, Canada

^10^Terrence Donnelly Centre for Cellular and Biomolecular Research, University of Toronto, Toronto, Ontario, Canada.

^11^Heart and Stroke/Richard Lewar Centre of Excellence, University of Toronto, Toronto, Ontario, Canada.

^12^Michael Smith Laboratories, School of Biomedical Engineering, University of British Columbia, Vancouver, British Columbia, Canada.

*Correspondence to [peter.zandstra@ubc.ca](mailto:peter.zandstra@ubc.ca), [m.radisic@utoronto.ca](mailto:m.radisic@utoronto.ca) & hxu@a2biotherapeutics.com

**Supplementary Figure 1.**Dimensions of well and microcantilever design within 96-well plate. (A) Dimensions of microcantilever geometries as shown in cross-sectional view. (B) Higher detail dimensions of individual well. (C) Total volume and surface area calculations for all compartments of well. (D) Beam bending formula for calculating force versus displacement curve. Deflection (δ), Force (F), Length of cantilever (L), Young’s Modulus (E), Moment of inertia (I), Radius of cylinder (r).

**
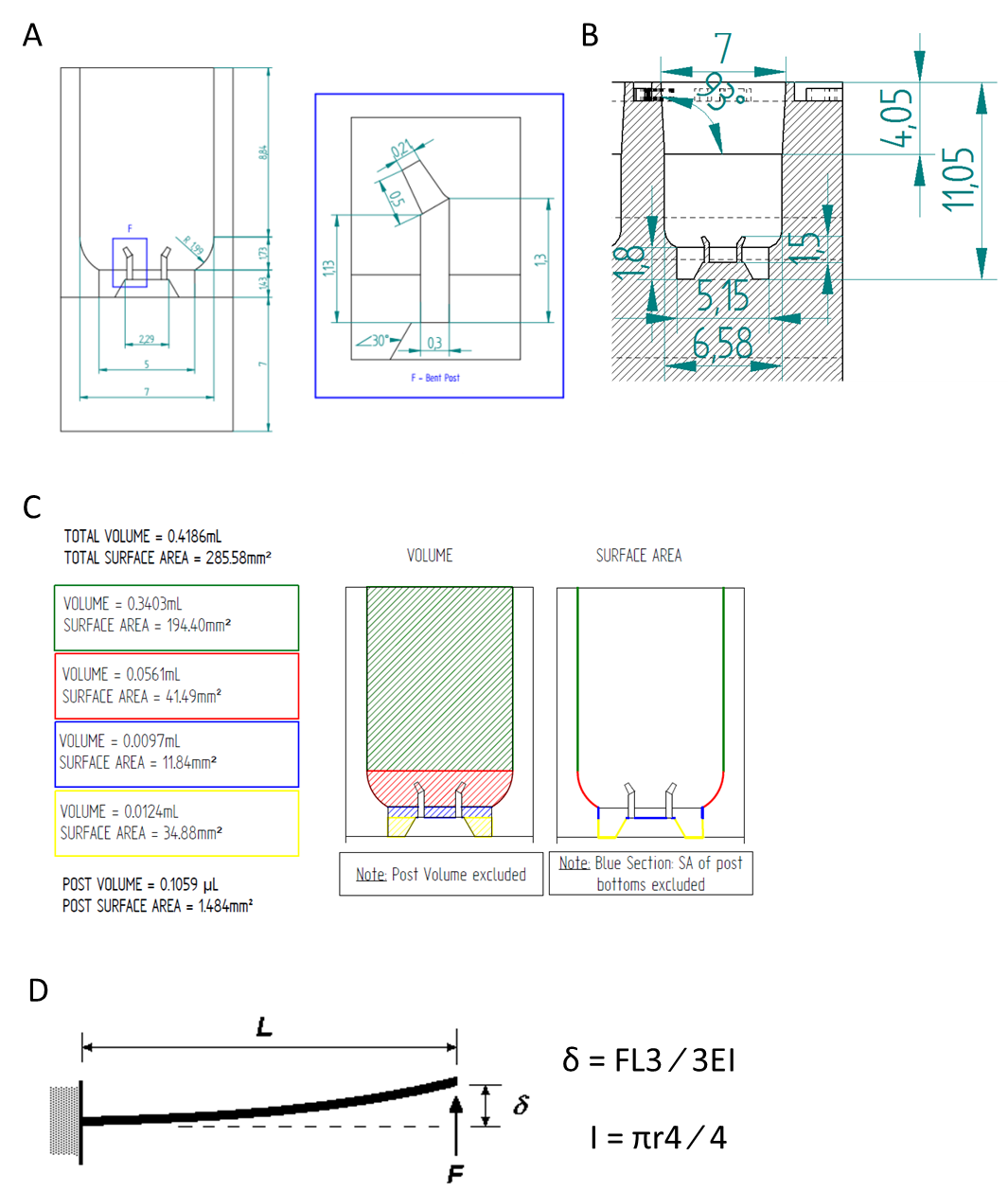
**

**Supplementary Figure 2.**Drafting of 3D printed mold shows decreased areas of stress during demolding process. (A) Green areas represent areas of high stress during demolding process, in contrast to yellow walls which represent low stress during demolding. Drafting angles into the walls convert green high stress areas into yellow low stress areas (left vs. right panels). (B) Schematics of angled walls show degree of drafting integrated into CAD design before 3D printing. Inset in blue box shows detailed dimensions of the drafted walls.


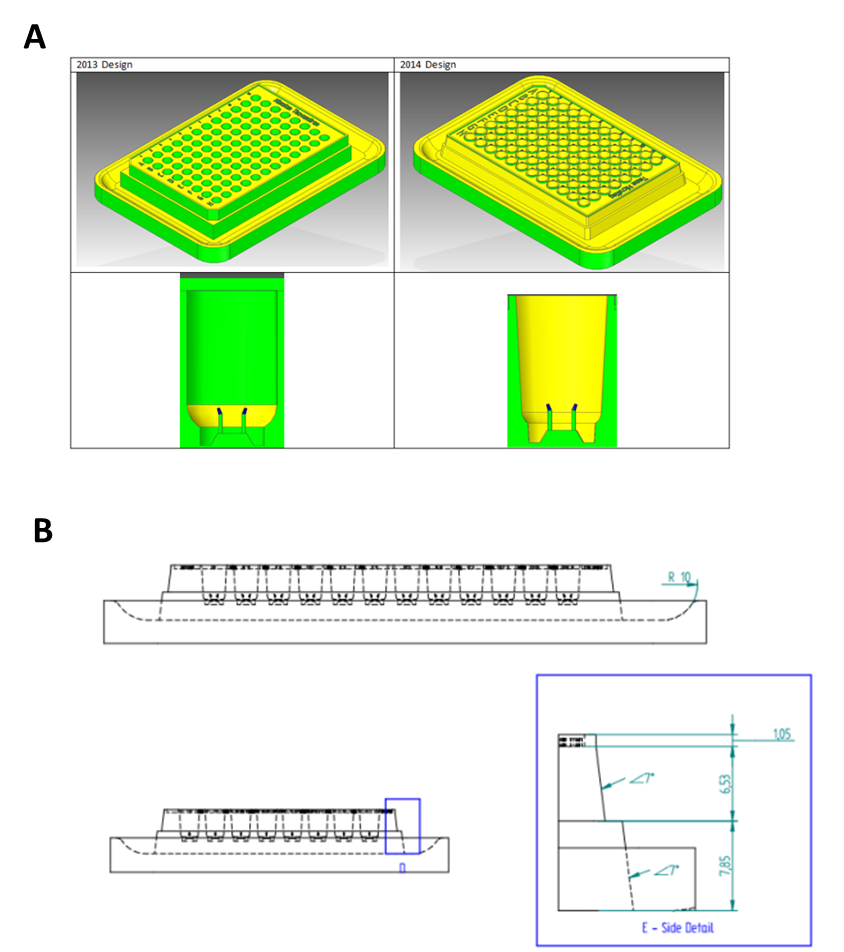


**Supplementary Figure 3.**Cell seeding of CaMiRi. (A) Seeding and tissue formation process flow. 12μl of cell-laden matrix are pipetted into the reservoir of each well, as shown in the first diagram. Polymerize in the incubator for 20 minutes. 200 uL of cell culture media is then slowly added into each well over top of the polymerized cell-laden collagen gel, as shown in the second diagram and return to the incubator. Over time the cells remodel around the two cantilevers as the cells start to form a compact tissue. (B) Time lapse imaging of remodeling CaMiRi composed of NKX2-5-GFP+ hPSC-CM. (C) Top and side view of cardiac microtissue on microcantilevers. CaMiRi in bright field shown on left, and in red (due to cell tracker dye) shown on right. Scale bars represent 2 mm.

**
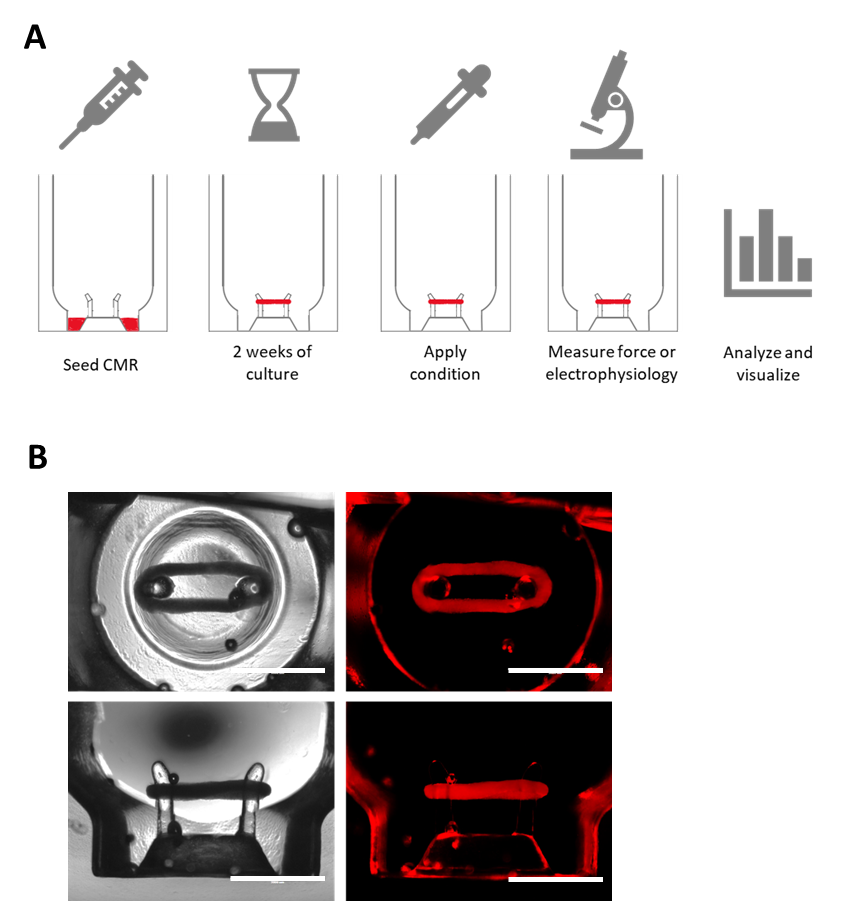
**

**Supplementary Figure 4.**Step-by step visual process of plate fabrication and cell seeding. (A) 3D printed positive mold of CaMiRi platform. (B) PDMS negative mold made of previous 3D printed mold. (C) PDMS positive molded from previous step. (D) Polyurethane negative master mold cast using PDMS positive mold from previous step. (E) PDMS CaMiRi plate molded from polyurethane master mold from previous step and autoclaved, ready to be coated and seeded. (F) Cell-laden collagen pipetted into wells over ice. (G) Excess cell-laden collagen on plate walls. (H) After gently tapping plate on flat surface, cell-laden collagen has completely sunk into the reservoirs to create an annulus of cell-laden collagen. (I) After 20 minutes in incubator, CaMiRi have polymerized. 200 µL of media is slowly pipetted into each well using a multichannel pipettor.

**
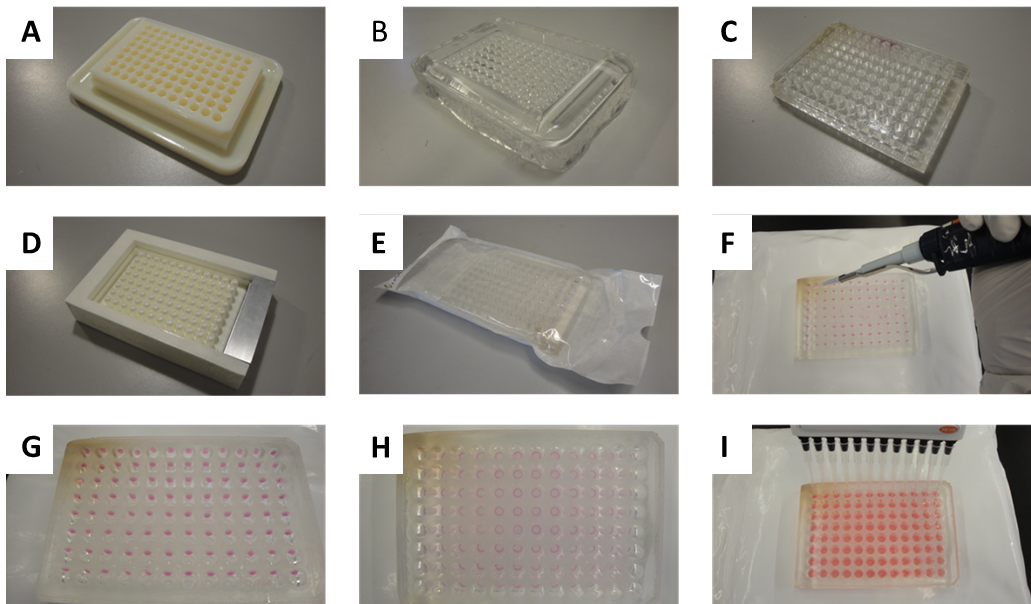
**

**Supplementary Figure 5.**Video capture and analysis of deflecting cantilevers. (A) Schematic depicting camera located above well capturing video of deflecting cantilever and edge detection (B) to calculate pixel movement during contractions and (C, D) batch processing of data using custom software designed by CellScale.

**
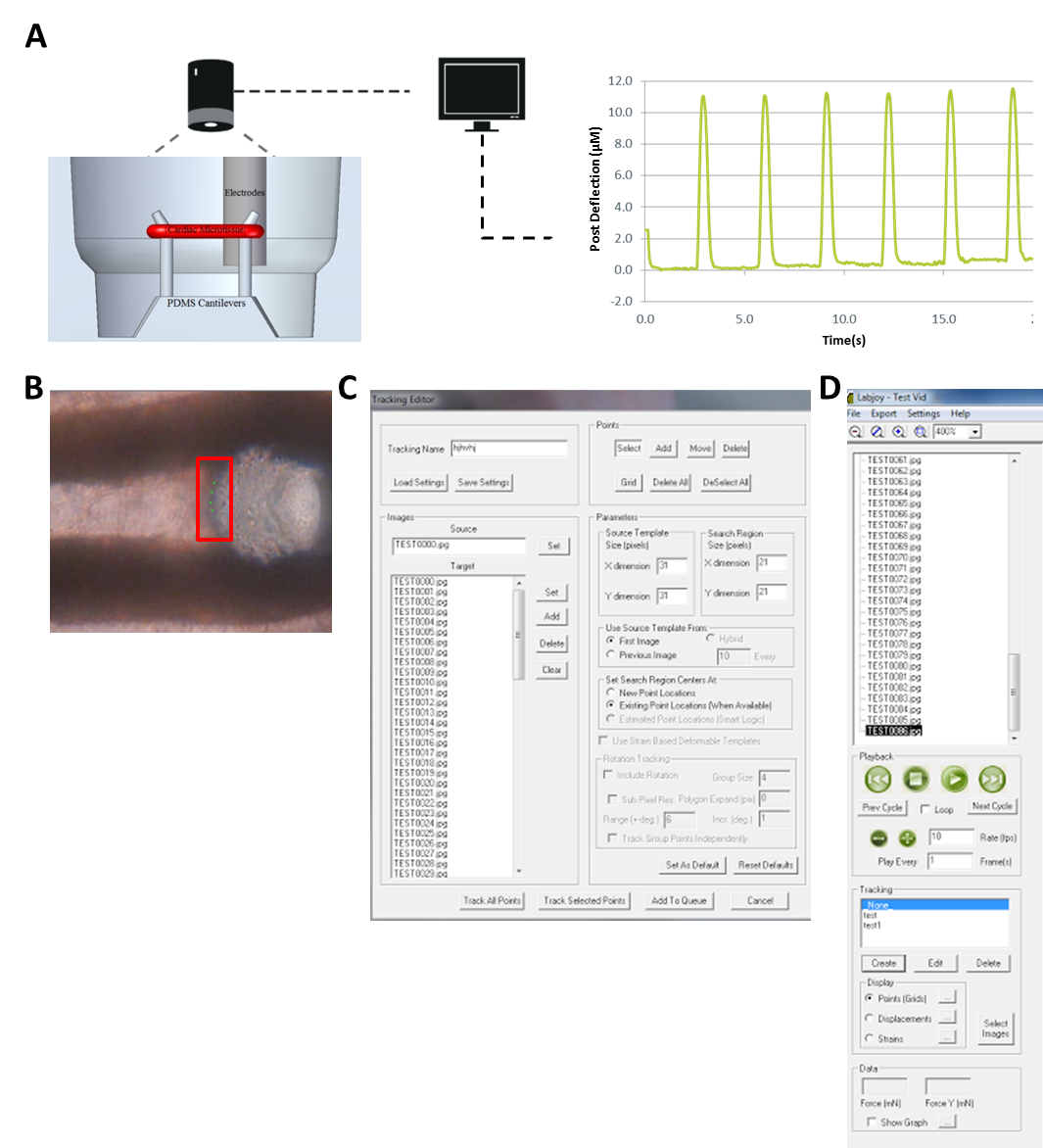
**

**Supplementary Figure 6.**Central Composite Design (CCD)-informed formulation of CaMiRi. (A, B) Legend and schematic of CCD parameters and conditions tested (low, medium, and high values) chosen for percentage of cardiomyocytes and cardiac fibroblasts in tissue (50%, 70%, 90% cardiomyocytes), collagen concentration (1.8, 2.0, and 2.2 mg/mL), and cell number per tissue (40,000; 70,000; 100,000 cells per tissue). (C) Contraction frequency of cardiac microtissues in all conditions measured in beats per minute show no significant differences between conditions. (D) Summary of surface functions of each combination of 3 factors tested in CCD study result in a predicted best-case formulation of 90% CM, 2.0 mg/mL collagen 1 concentration, and 75,000 cells per CaMiRi.

**
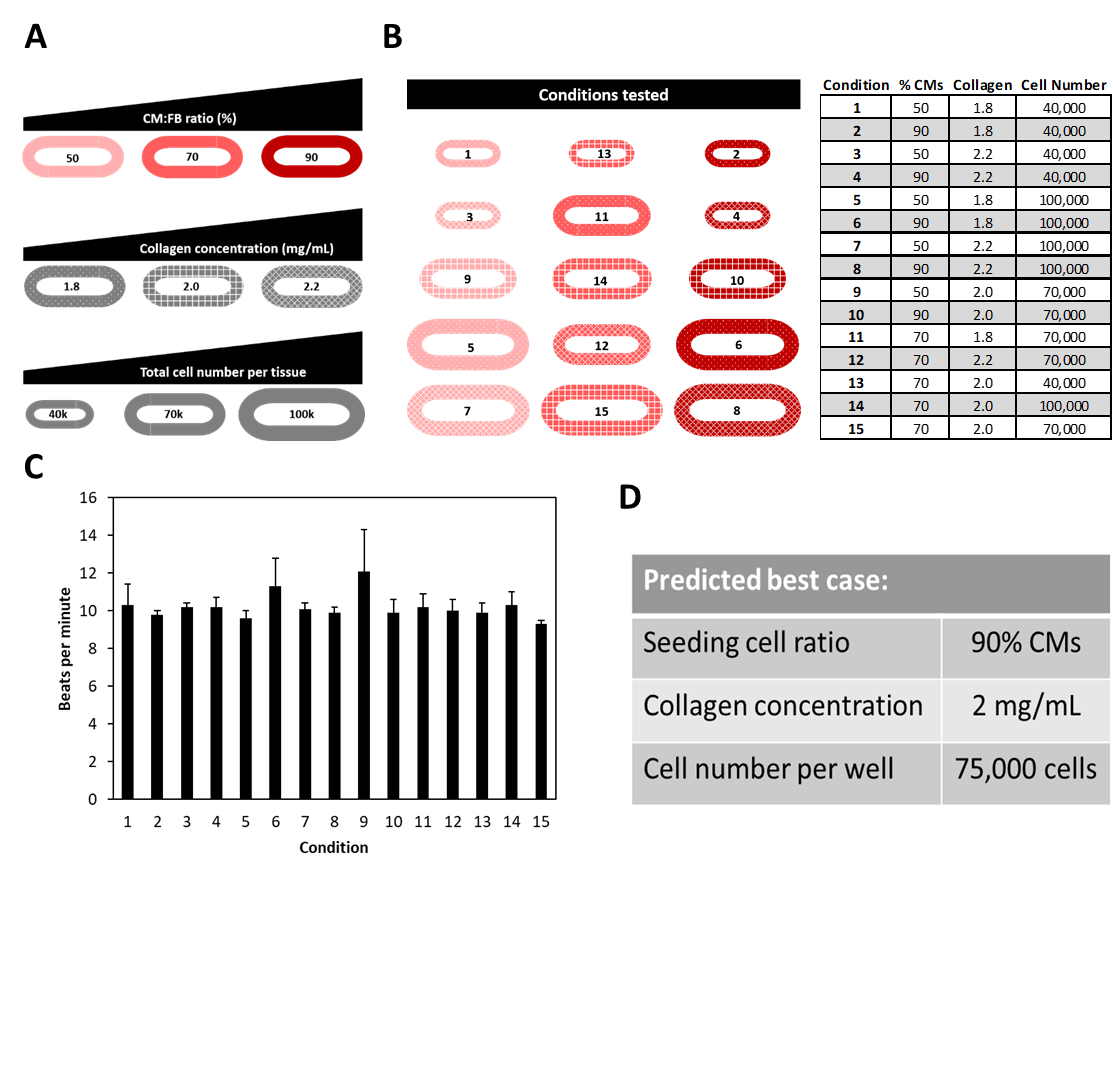
**

**Supplementary Figure 7.**Central Composite Design (CCD)-informed formulation of CaMiRi. (A) Schematic of CCD parameters (low, medium, and high values) chosen for collagen concentration (1.8, 2.0, and 2.2 mg/mL), cell number per tissue (40,000; 70,000; 100,000 cells per tissue), and percentage of cardiomyocytes and cardiac fibroblasts in tissue (50%, 70%, 90% cardiomyocytes). (B) Surface functions of each combination of 3 factors tested in CCD study resulting in a predicted best case formulation of 90% CM, 2.0 mg/mL collagen 1 concentration, and 75,000 cells per CaMiRi.

**
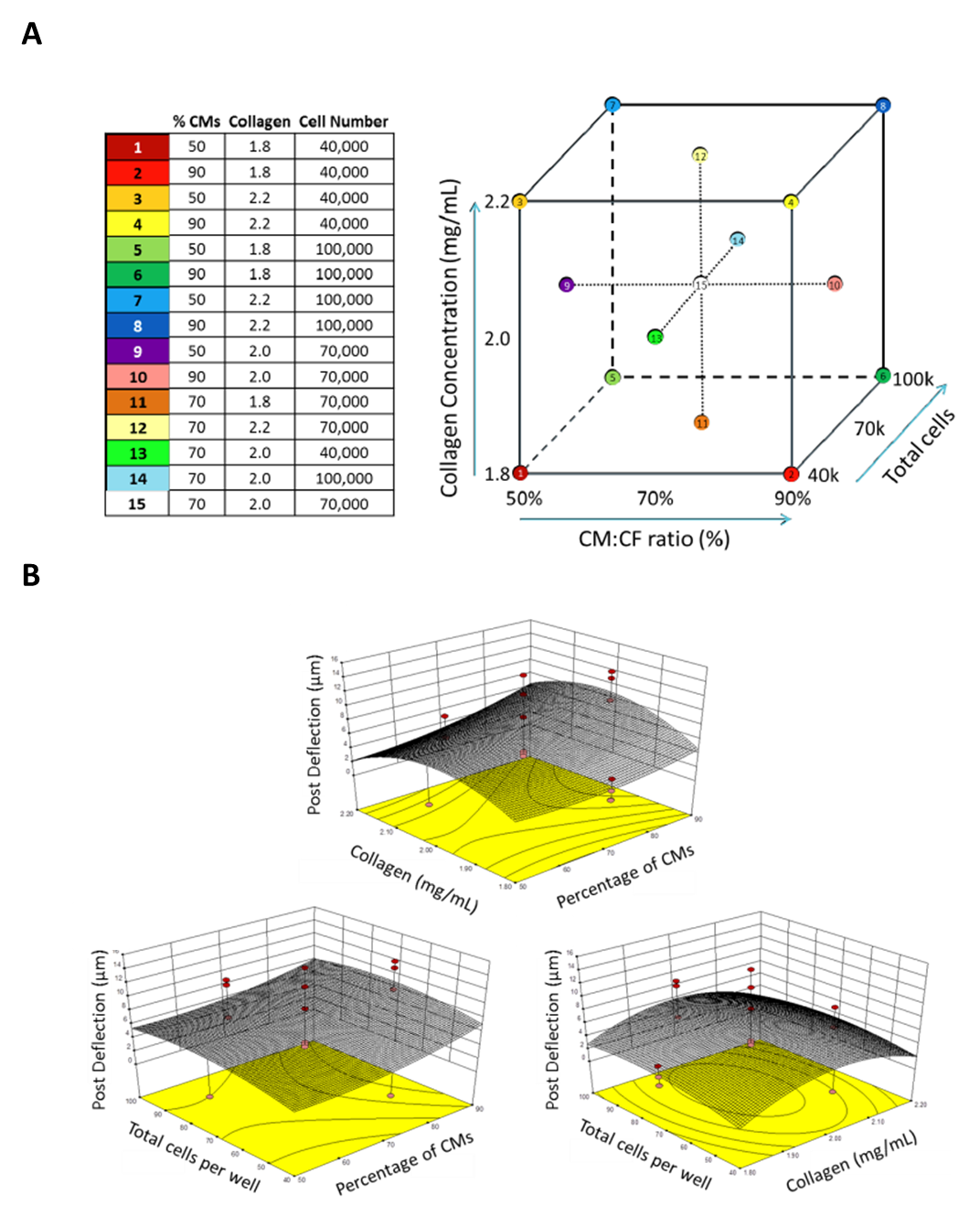
**

**Supplementary Figure 8.**Electrophysiology-based assay using CaMiRi. (A, B) Electrophysiological assessment of maximum capture rate (a measure of tissue response to stimulation), and excitation threshold (a measure of tissue excitability), of growth factor effects on cardiac microtissues over 2 weeks. IGF-1 and HRG show improved excitation threshold in cardiac microtissues. Data are reported from three experiments with at least two replicates each, as the mean ± standard deviation. *P < 0.05.

**
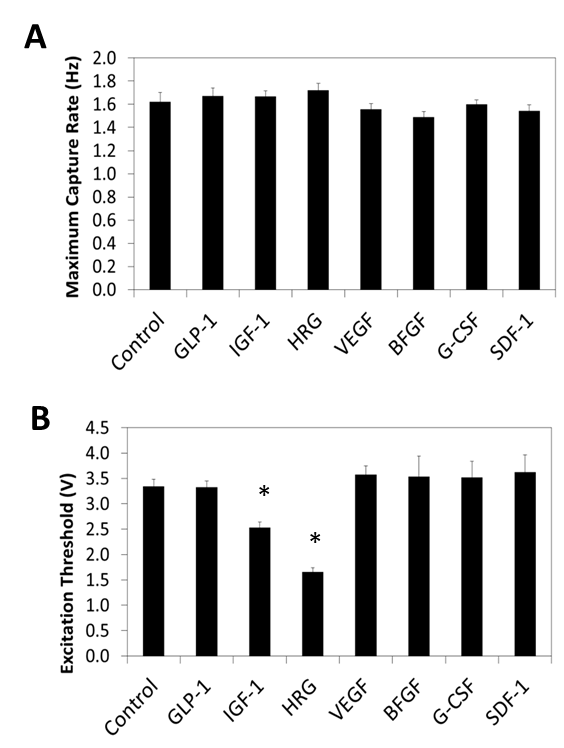
**

**Supplementary Figure 9.**Automated seeding of CaMiRi. (A) 4x 2.5µL drops of cellular mixture were pipetted to N, E, S, and W positions inside each well. (B) 16 wells were seeded at a time, in 6 iterations to cover the 96 well plate, using an Agilent Bravo automated liquid handler. (C) Discrete droplets post-dispense (left) were driven into contiguous rings (right) following cooled centrifugation for 5 minutes at 200 x g. (D) Centrifugation conditions were optimized functionally by measuring CaMiRi contraction amplitude 14 days post-seeding. (E) 96 well plate of CaMiRi, 14 days after automated seeding.

**
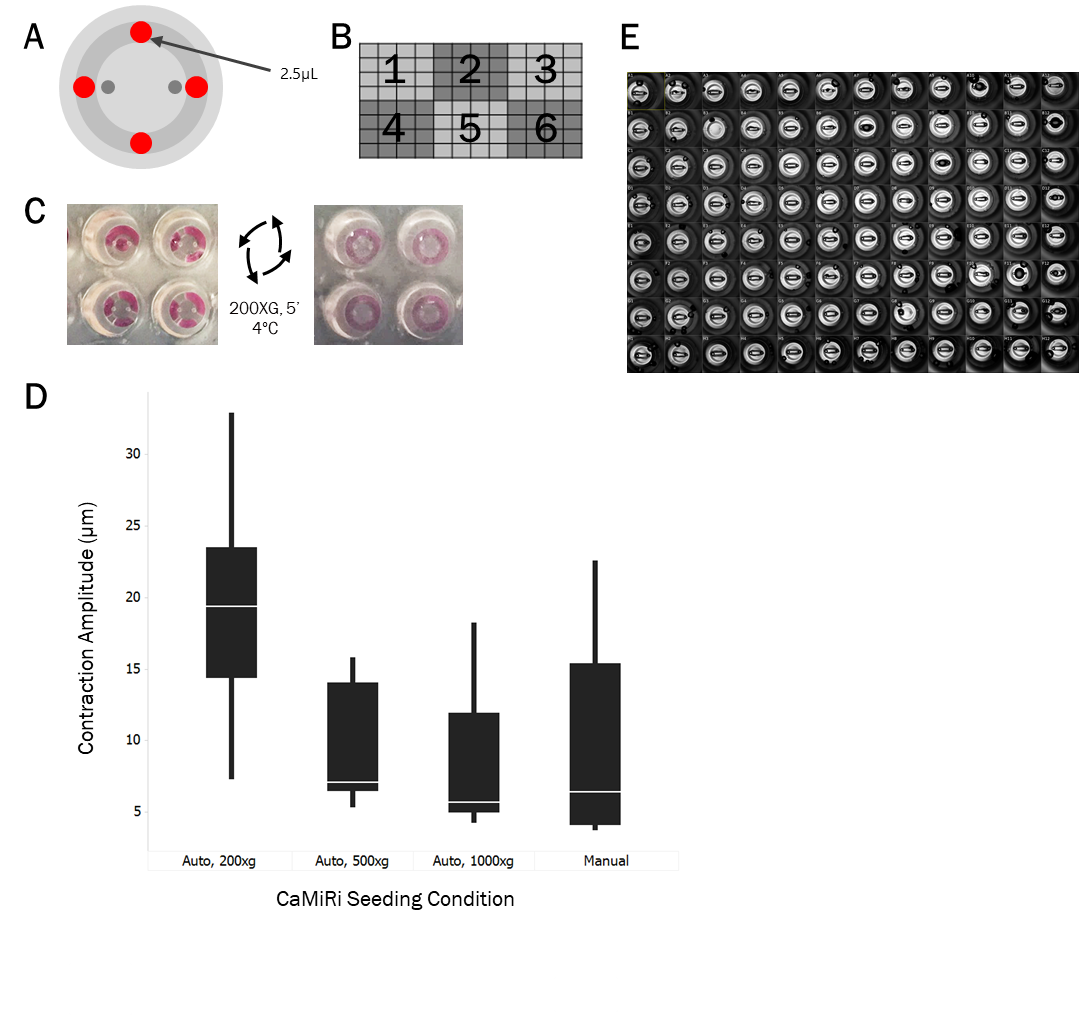
**

**Supplementary Figure 10.**Layered multicellular CaMiRi. (A) Using a layering approach via sequential seeding, multicellular tissues can be generated using the CaMiRi platform. Cell type 1 (green) can be seeded first, permitted to remodel, and then cell type 2 (red) can be seeded subsequently to create an additional outer layer.

**
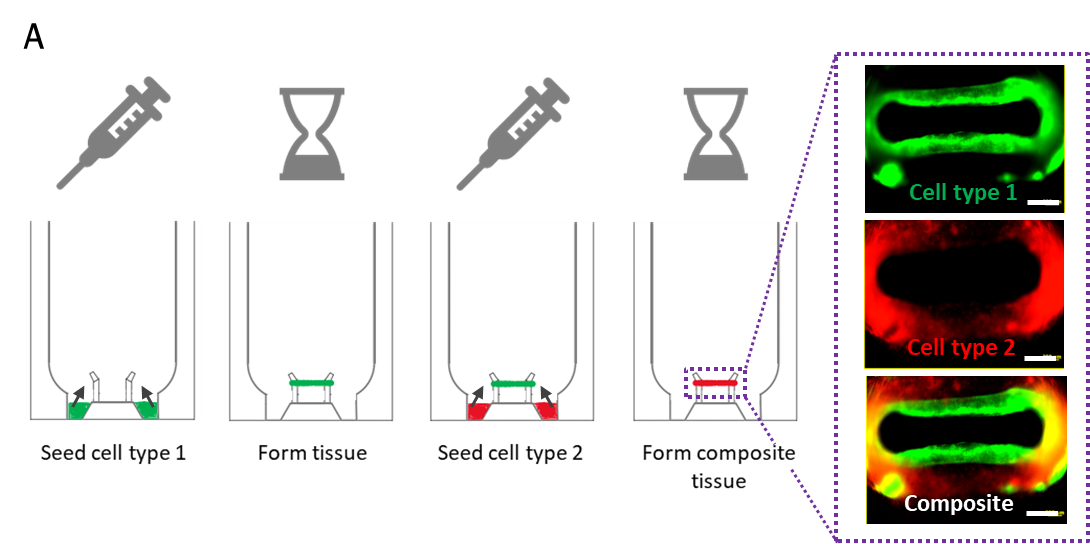
**

**Supplementary Video 1.**Self-organized CaMiRi in a well of 96-well plate on day 14. Cantilevers can be seen deflecting inward in response to the contractile force exerted by the cardiac microtissues.
